# Association of subgingival Epstein–Barr virus and periodontitis

**DOI:** 10.12688/f1000research.52624.1

**Published:** 2021-05-24

**Authors:** Chaerita Maulani, Sri Lelyati C. Masulili, Widayat Djoko Santoso, Nurtami Soedarsono, Lindawati Kusdhany, Elza Ibrahim Auerkari

**Affiliations:** 1 Faculty of Dentistry, Universitas Indonesia, Jakarta, 10430, Indonesia; 2 Department of Periodontology, Faculty of Dentistry, Universitas Indonesia, Jakarta, 10430, Indonesia; 3 Department of Internal Medicine, Faculty of Medicine, Universitas Indonesia, Jakarta, 10430, Indonesia; 4 Department of Oral Biology, Faculty of Dentistry, Universitas Indonesia, Jakarta, 10430, Indonesia; 5 Department of Prosthodontics, Faculty of Dentistry, Universitas Indonesia, Jakarta, 10430, Indonesia

**Keywords:** Epstein-Barr Virus, Periodontitis, Gingival Crevicular Fluid

## Abstract

**Background:** The Epstein–Barr virus (EBV) is gaining interest as a possible agent in the etiology of periodontitis. Previous studies have shown controversy on whether EBV DNA in the subgingival periodontal pockets is associated with periodontitis. The aim of the present study was to seek the potential relationship between EBV and periodontitis.

**Methods:** Data on socio-demographics, oral health, and periodontal health were recorded, and samples were collected from gingival crevicular fluid, using sterile paper point. This case–control study of 118 participants included 59 subjects with severe periodontitis and 59 control subjects with mild periodontitis. The EBV load was determined by quantitative real-time PCR.

**Results:** EBV DNA was detected in 37.3% of the case samples and in 18.6% of the control samples. There was no significant difference in the load of EBV DNA between severe and mild periodontitis (p>0.05). The observed load of EBV DNA was up to 4.55x10 ^5^ copies/mL. The detected EBV DNA was significantly associated with the plaque index and the oral hygiene index (all p<0.05).

**Conclusions:** A significant association was not found, but EBV might contribute to periodontitis. Gingival crevicular fluid is useful for monitoring the EBV load by the real-time PCR technique.

## Introduction

Periodontal diseases consist of chronic inflammatory conditions that affect the supporting tissue of the teeth. Localized inflammation within the gingiva caused by microorganisms in the dental plaque leads to gingivitis. Untreated gingivitis can progress to chronic periodontitis with the advanced loss of gingiva, bone and periodontal ligament. ^1^ The periodontitis process is a multifactorial interplay between microbial, host and environmental factors. In the development of periodontitis, microbial agents are the important key. However, microbial communities in periodontitis compared with healthy individuals showed that the presence of bleeding was not associated with a specific microbiome although total bacterial load tends to be higher in bleeding sites. ^2^ Periodontal breakdown is site specific, and can be explained not just by bacterial specificity or immunopathology, but also by a combined herpesvirus–bacterial infection. ^3^ With this contribution, herpesvirus has emerged as a significant periodontal pathogen. ^4^ Epstein–Barr virus (EBV) is one of eight herpesviruses that can cause disease in humans. EBV is found in more than 90% of the world populations with oral transmission as the primary route of the EBV infection. ^5^

However, variable findings have been shown on the presence of EBV in subgingival pockets. Some studies have reported higher prevalence of EBV DNA with increasing severity of periodontitis ^6^^–^^9^ while others have shown weak or no relationship between them. ^10^^–^^12^ For these purposes, presence of EBV DNA was determined by techniques such PCR, ^13^ nested PCR ^6^^,^^7^ or quantitative real-time PCR. ^9^^,^^11^

The aim of this study was to examine using quantitative real-time PCR whether EBV DNA in subgingival pockets has higher prevalence in systemically healthy patients with severe periodontitis than in those with mild periodontitis.

## Methods

### Study population

This case–control study included 118 systematically healthy, 18 to 66 years old individuals with at least 14 erupted teeth ^12^ and with either healthy periodontal condition, gingivitis or periodontitis. Subjects were excluded if they had systemic disease, periodontal treatment in the last six months, pregnancy, lactation, used antibiotic therapy or immunosuppressants in the last 3 months, had acute illness, detectable inflammation within oral mucosa, or refused to take part in the research. All participants were given a thorough verbal and written explanation of the study and were required to sign an informed consent form. The study was approved by the Institutional Review Board, protocol number 070390418. The study was conducted within three different district offices in Central Jakarta between June 2018 and March 2019. The case and control subjects were recruited from the same districts. The subjects not meeting the inclusion criteria were excluded and the final study consisted of 59 case patients with severe periodontitis and 59 controls with mild periodontitis. The sample size was considered acceptable in comparison to previous studies. ^9^^,^^13^ The sample size also give 80% power with the effect size 0.3 (medium) with a 0.05 two tails significance level.

### Clinical periodontal examination

Clinical periodontal examinations were performed by two investigators (CM, PW) for each subject with measurement of plaque index (PI), ^14^ bleeding on probing (BOP), pocket depth (PD), clinical attachment level (CAL), and oral hygiene index–simplified (OHIS). ^15^^,^^16^ The plaque index was measured at four sites per tooth according to Loe. ^14^ The UNC-15 probe (Hu-Friedy, USA) was used to measure PD at six sites per tooth (disto-buccal, mid-buccal, mesio-buccal, disto-lingual, mid-lingual and mesio-lingual). The distance between the cementoenamel junction and the base of the periodontal pocket was used to measure clinical attachment loss. BOP was measured at one site per tooth using papillary bleeding index by Mühlemann. ^15^ OHIS was recorded by addition debris index and calculus index, measured at two sites per tooth. ^16^

Patient grouping was according to classification of periodontal and peri-implantitis condition. ^17^ Stage 3 and stage 4 periodontitis cases with CAL ≥5 mm and PD ≥6 mm were included in the severe periodontitis case category. The control group included gingivitis, stage 1 and stage 2 periodontitis cases of CAL <5 mm and PD ≤5 mm. ^15^^,^^18^

The examiner reliability was verified with interclass correlation coefficient (ICC) of 0.741 (95% CI 0.507–0.846) and 0.837 (95% CI 0.784–0.875), indicating good and excellent agreement for PD and CAL respectively. The kappa value for PI and BOP ranged from 0.737 to 0.805, indicating substantial agreement.

Gingival crevicular fluid (GCF) was collected from four sites of the deepest periodontal pocket and randomly at one site of each quadrant in healthy sulcus. Saliva and supragingival plaque were cleaned gently with sterile cotton pellets, separated with cotton rolls, and air dried before sampling. A sterile paper point ISO 30 (Roeko, Germany) was inserted gently into the periodontal pocket until it reached the base of pocket depth with mild resistance and held for about 30 seconds. Bleeding sites were carefully avoided. The collected samples were pooled then put in a plastic tube with 1 mL of sterile phosphate buffered saline. The vial was vortexed for about 1 min and then centrifuged for approximately 20 min at 3000 rpm. The supernatants were collected and samples stored at −20°C until further processing.

### DNA extraction and real-time PCR

After centrifugation, DNA was extracted from GCF using a commercial kit (QIAamp DNA Mini Kit, Qiagen, Germany) according to the instructions. The presence and quantity of extracted DNA was tested by NanoQuant Infinite ^®^ M200Pro. The real-time PCR method was used to determine the amount of EBV DNA in the samples. The analysed amount of EBV DNA in each sample was in a volume of 10 μL. The single copy gene encoding the EBV nuclear antigen 1 (EBNA1) was the target sequence (EBNA1). Real-time PCR LightCycler ^®^ 480 II (Roche Diagnostics GmbH, Mannheim, Germany) was used with a commercial kit (EBV/ISIN/100, GeneProof, Brno, CZ). The kit has four standards containing EBV calibrator from 10 ^1^ to 10 ^4^ copies of EBNA1 per μL. From a linear regression to standard curves, quantification was conducted by extrapolation. The PCR conditions were set as directed by the manufacturer.

### Statistical analysis

The findings were analyzed using IBM SPSS v.23 (RRID:SCR_019096), for which an open-access alternative would be JASP ( https://jasp-stas.org; RRID:SCR_015823) To determine the association between EBV and periodontitis, the Chi-squared test was used. Statistical significance was assumed at a p-value of 0.05 or less.

## Results

In total, 118 subjects with mild-to-severe periodontitis, ranging from 18 to 66 years old, mean 38.15 ± 14.4 years, were evaluated for periodontal status including EBV DNA in gingival crevicular fluid. Table 1 shows the demographic and clinical periodontal parameters.

**Table 1.** Demographic data and clinical parameters.

Demographic data	Case (n = 59)	Control (n = 59)	p-value
Age (years) ^a^, median (IQR)	49 (19)	24 (14)	0.000**
Male ^b^, n(%)	19 (27.5)	50 (72.5)	0.000**
Female, n(%)	40 (81.6)	9 (18.4)	
Non-smokers ^b^, n(%)	43 (58.1)	31 (41.9)	0.036*
Smokers, n(%)	16 (36.4)	28 (63.6)	
Clinical parameters			
PD (mm) ^a^, median (IQR)	1.87 (0.63)	1.44 (0.33)	0.000**
CAL (mm) ^a^, median (IQR)	2.60 (1.25)	1.48 (0.42)	0.000**
BOP, mean ± SD ^b^	1.47 ± 0.90	0.77 ± 0.62	0.000**
Plaque index ^a^, median (IQR)	1.24 (0.57)	0.88 (0.72)	0.001*
OHIS ^a^, median (IQR)	2.19 (1.25)	1.68 (1.28)	0.026*
Number of teeth ^a^, median (IQR)	24 (6)	27 (2)	0.000**

^a^ Mann–Whitney test.

^b^ Continuity correction.

^c^ Independent t-test

PD: pocket depth, CAL: clinical attachment level, BOP: bleeding on probing, PI: plaque index, OHIS: oral hygiene index simplified.

The EBV DNA was detected in 28.0% of all subjects, in 37.3% of cases of severe periodontitis, and in 18.6% of the control cases of mild periodontitis. The highest EBV DNA level was 4.55 × 10 ^5^ copies/mL in severe periodontitis. No statistically significant difference in the detected EBV DNA was found between the case and control groups ( Table 2).

**Table 2.** Prevalence of EBV DNA in GCF samples.

Variable	Case (n = 59)	Control (n = 59)	p-value
EBV ^a^, n (%)	22 (66.7)	11 (33.3)	0.086

^a^ Mann–Whitney test.

In Table 3, clinical parameters of periodontitis were compared with EBV DNA detection. It showed that the EBV DNA load was significantly associated with the plaque index and OHIS, but not with age, gender and other clinical parameters of periodontitis. Although also not statistically significant, more EBV DNA was detected from subjects with severe periodontitis than from those with mild periodontitis.

**Table 3.** Demographic data and clinical parameters in relation to detected EBV DNA.

Variable	Detected (n = 33)	Not detected (n = 85)	p-value
Age (years) ^a^, median (IQR)	35 (26)	36 (28)	0.990
Male ^a^, n (%)	24 (34.8)	45 (65.2)	0.080
Female ^a^, n (%)	9 (18.4)	40 (81.6)	
PD ^a^, median (IQR)	1.67 (0.71)	1.59 (0.53)	0.159
CAL ^a^, median (IQR)	2.03 (1.42)	1.86 (1.13)	0.078
BOP ^a^, median (IQR)	1.11 (1.01)	0.89 (1.07)	0.337
PI, mean ± SD ^b^	1.29 ± 0.44	1.04 ± 0.52	0.002*
OHIS ^a^, median (IQR)	2.39 (1.06)	1.70 (1.44)	0.001*
Number of teeth ^a^, median (IQR)	26 (6)	26 (5)	0.765

^a^ Mann–Whitney test.

^b^ Independent t-test

PD: pocket depth, CAL: clinical attachment level, BOP: bleeding on probing, PI: plaque index, OHIS: oral hygiene index simplified.

## Discussion

The etiology of periodontal disease was previously thought to be bacteria driven, but later studies have suggested that herpesviruses, especially EBV, are also involved. ^19^^,^^20^ This hypothesis emerged because some periodontal disease cannot be explained by bacterial activity alone. ^4^ The results of our study do not indicate a role for EBV to play in periodontitis, similarly as in findings made in some previous studies. ^10^^,^^12^ In cases of severe periodontitis, the prevalence of EBV detected at periodontal pockets was about 37.3%. This was only slightly lower than in a previous study in India with 44.2% in aggressive periodontitis ^9^ but clearly lower than observed values for chronic periodontitis: 72.5% in Iran; ^6^ 74.49% in Indonesia; ^21^ and 66–68% in Japan. ^7^^,^^8^ The differences in terms of detected EBV DNA between the cases or severe periodontitis and the controls of mild periodontitis were not statistically significant, but showed significant association with the clinical indicators of plaque index and OHIS.

Age, gender and smoking status were associated significantly with periodontitis, so were the clinical parameters of periodontitis ( Table 1). The results are in line with a previous study that smoking has been determined in periodontal classification as an important risk factor in periodontitis. ^18^

The detected EBV DNA was not significantly associated with periodontitis, age, gender and clinical parameters (pocket depth, clinical attachment level, bleeding on probing) of periodontitis (p > 0.05). However, the increasing plaque index and OHIS were significantly associated with detected EBV DNA ( Table 3). This is in agreement with the findings of a previous study. ^13^

Low rates of EBV detection could have various reasons including study methodology. In the present study, the periodontitis group had a mean of clinical attachment level of 2.74 ± 0.92, while in other studies, a mean clinical attachment level of 6.7 ± 1.6 mm did not indicate association between prevalence of EBV and periodontitis. ^12^ Similarly, the probing depth was 1.9 ± 0.52 mm, well below of the 5.8 ± 1.2 mm in the comparison study.

We analysed the presence of EBV DNA by sterile paper points in 118 patients from gingival crevicular fluid (GCF) as done previously using paper strips. ^13^ In other studies, EBV DNA has been detected from subgingival plaque with sterile paper point ^7^^,^^8^^,^^12^^,^^21^ or curettage. ^6^^,^^10^^,^^22^ GCF can only be obtained in small amounts in healthy sulcus, but during inflammation, GCF flow increases 5.5-fold as shown in experimental gingivitis. ^23^ The resting void volume in GCF is highly dependent upon the pocket depth; as a pocket increases from 3 to 6 mm, the resting void volume increases by 50%. ^24^ When compared with baseline, the GCF level in periodontitis decreased after 6 weeks posttreatment of phase-I periodontal therapy. ^13^ Although GCF samples could be one reason for relatively low detection rates, subgingival plaque with curette technique can also infrequently detect EBV DNA that was not correlated with increased probing depth in another study. ^11^ A proposed mechanism for the EBV role suggests that periodontal pockets harbour EBV and contain excessive reactive oxygen species, which induce excessive development of receptor activator of nuclear factor κB ligand (RANKL) that activates osteoclasts. ^25^

The lowest positively detected EBV DNA was indicated at 3.4 copies/mL. The range for the controls or mild periodontitis patients was up to 7.98 × 10 ^4^ copies/mL and for the severe periodontitis cases up to 4.55 × 10 ^5^ copies/mL. In a previous study in Japan on chronic periodontitis, the observed upper range of detected EBV DNA was about 10 ^3^–10 ^9^ copies/mL and an order of magnitude higher in deep pockets (PD ≥ 5 mm) than in shallower pockets (PD ≤3 mm). ^8^

Another reason for variability is the grouping of periodontitis that does not present a good contrast between cases and controls when healthy subjects are uncommon, so that subjects with gingivitis and mild periodontitis provide the control group. As the recent classification of periodontitis does not differentiate between aggressive and chronic periodontitis, the severity of periodontitis is based on staging. ^18^

Furthermore, sensitive methods like nested PCR ^6^^,^^7^ to identify EBV DNA have the risk of overestimating the result, while other methods like automated real-time PCR assays in previous studies found EBV DNA less frequently, ^10^^–^^12^ including in our investigation.

A previous study about EBV in another Indonesian population of Bandung, West Java, found all subjects seropositive for EBV with 75% detection rate in subgingival EBV DNA using the same technique of qRT-PCR. ^21^ Some differences in environmental, ethnic and immunogenetic factors may contribute, but are not expected to be large. Limitations of the study include the relatively small sample size and the unclear difference between the detection rates of EBV DNA from GCF samples and indicated high seroprevalence for EBV in the previous study. ^21^

## Conclusions

Our findings showed no significant association between EBV and periodontitis, although in cases of severe periodontitis EBV DNA was detected more frequently and at higher maximum level than in cases of mild periodontitis. Gingival crevicular fluid is useful to check the EBV load using a real-time PCR technique.

## Data availability

### Underlying data

Harvard Dataverse: Underlying data for ‘Association of subgingival Epstein–Barr virus and periodontitis’. https://doi.org/10.7910/DVN/AR3Y00

This project contains the following underlying data: F1000_EBV_RawData_EIA_2021.csv

Data are available under the terms of the Creative Commons Zero “No rights reserved” data waiver (CC0 1.0 Public domain dedication).

## Consent

Written informed consent for publication of the patient details was obtained from the patients.

## Author contributions

CM: Data Curation, Formal Analysis, Investigation, Visualization, Writing-Original Draft Preparation; SLM: Conceptualization, Project Administration, Supervision; WDS: Methodology, Supervision, Validation; NS: Validation, Visualization; LK: Formal Analysis, Validation; EIA: Conceptualization, Funding Acquisition, Methodology, Supervision, Writing-Review & Editing.
