# Association of subgingival Epstein–Barr virus and periodontitis

**DOI:** 10.12688/f1000research.52624.2

**Published:** 2021-06-28

**Authors:** Chaerita Maulani, Sri Lelyati C. Masulili, Widayat Djoko Santoso, Nurtami Soedarsono, Lindawati Kusdhany, Elza Ibrahim Auerkari

**Affiliations:** 1 Faculty of Dentistry, Universitas Indonesia, Jakarta, 10430, Indonesia; 2 Department of Periodontology, Faculty of Dentistry, Universitas Indonesia, Jakarta, 10430, Indonesia; 3 Department of Internal Medicine, Faculty of Medicine, Universitas Indonesia, Jakarta, 10430, Indonesia; 4 Department of Oral Biology, Faculty of Dentistry, Universitas Indonesia, Jakarta, 10430, Indonesia; 5 Department of Prosthodontics, Faculty of Dentistry, Universitas Indonesia, Jakarta, 10430, Indonesia

**Keywords:** Epstein-Barr Virus, Periodontitis, Gingival Crevicular Fluid

## Abstract

**Background:** The Epstein–Barr virus (EBV) is gaining interest as a possible agent in the etiology of periodontitis. Previous studies have shown controversy on whether EBV DNA in the subgingival periodontal pockets is associated with periodontitis. The present study aimed to seek the potential relationship between EBV and periodontitis.

**Methods:** Samples were taken from gingival crevicular fluid using sterile paper points, and data on sociodemographics, oral health, and periodontal health were recorded. This case-control study of 118 participants included 59 subjects with severe periodontitis and 59 control subjects with mild periodontitis. Quantitative real-time PCR was used to determined EBV load.

**Results:** EBV DNA was detected in 37.3% of the case samples and 18.6% of the control samples. There was no significant difference in a load of EBV DNA between severe and mild periodontitis (p>0.05). The observed load of EBV DNA was up to 4.55x10 ^5^ copies/mL. The detected EBV DNA was significantly associated with the plaque index and the oral hygiene index (p<0.05).

**Conclusions:** Although no significant association was found, EBV may play a role in periodontitis. The real-time PCR methods can be used to monitor the EBV load in gingival crevicular fluid.

## Introduction

Periodontal diseases consist of chronic inflammatory conditions that affect the supporting tissue of the teeth. Localized inflammation within the gingiva caused by microorganisms in the dental plaque leads to gingivitis. Untreated gingivitis can progress to chronic periodontitis with the progressive loss of gingiva, bone, and periodontal ligament ^1^. The periodontitis process is a multifactorial interplay between microbial, host, and environmental factors. In the development of periodontitis, microbial agents are the critical key. However, microbial communities in periodontitis compared with healthy individuals showed that bleeding was not associated with a specific microbiome, although total bacterial load tends to be higher in bleeding sites ^2^. Periodontal breakdown is site-specific. It can be explained not just by bacterial specificity or immunopathology but also by a combined herpesvirus–bacterial infection ^3^. With this contribution, herpesvirus has emerged as a significant periodontal pathogen ^4^. Epstein–Barr virus (EBV) is one of eight herpesviruses that can cause disease in humans. EBV is prevalent in about 90% of the world’s population, with oral transmission being the most common route of infection ^5^.

However, the presence of EBV in subgingival pockets has yielded mixed results. Some studies have reported a higher prevalence of EBV DNA with increasing severity of periodontitis ^6– 9^, while others have shown weak or no relationship ^10– 12^.

Oral cavity samples, such as saliva, can be used to determine the presence and quantity of viral DNA. EBV is commonly present in the saliva of periodontitis patients ^13, 14^. Subgingival plaque, gingival crevicular fluid (GCF), and gingival tissue can all be used to detect EBV in the periodontal environment ^7, 15, 16^. The study used GCF as a sampling method because it resembles normal serum in the periodontal environment and allows multiple sampling sites within the oral cavity ^17^. GCF sampling can be collected noninvasively using paper points and is an ideal tool to detect host-microorganism interactions and the severity of periodontal inflammations ^18^. The previous study about EBV detection from GCF show significant results between periodontitis and control ^15^. The presence of EBV DNA can be analyzed using PCR ^15^, nested PCR ^6, 7^ or quantitative real-time PCR ^9, 11^.

This study aimed to examine using quantitative real-time PCR whether EBV DNA in subgingival pockets has a higher prevalence in systemically healthy patients with severe periodontitis than in those with mild periodontitis.

## Methods

### Study population

This case-control study included 118 systematically healthy, 18 to 66 years old individuals with at least 14 erupted teeth ^12^ and with either healthy periodontal condition, gingivitis or periodontitis. There were about 140 subjects, and 118 of them were willing to participate in the study. Subjects were excluded if they had systemic disease, periodontal treatment in the last six months, pregnancy, lactation, used antibiotic therapy or immunosuppressants in the last 3 months, had an acute illness, detectable inflammation within oral mucosa, or refused to take part in the research. All participants were given a thorough verbal and written explanation of the study and were required to sign an informed consent form. The study was approved by the Institutional Review Board, protocol number 070390418. Between June 2018 and March 2019, the research was conducted at three different district offices in Central Jakarta. The case and control subjects were recruited from the same districts. The subjects who did not meet the inclusion criteria were excluded, leaving 59 case patients with severe periodontitis and 59 controls with mild periodontitis in the final study. The sample size was considered acceptable in comparison to previous studies ^9, 15^. The sample size also gives 80% power with the effect size 0.3 (medium) with a 0.05 two-tails significance level.

### Clinical periodontal examination

For each subject, two investigators (CM, PW) performed periodontal examinations, measuring plaque index (PI) ^19^, bleeding on probing (BOP), pocket depth (PD), clinical attachment level (CAL), and oral hygiene index–simplified (OHIS) ^20, 21^. The plaque index was measured at four sites per tooth, according to Loe ^19^. The UNC-15 probe (Hu-Friedy, USA) was used to measure PD at six sites per tooth (disto-buccal, mid-buccal, mesio-buccal, disto-lingual, mid-lingual and mesio-lingual). The clinical attachment loss was determine by distance between the cementoenamel junction and the base of the periodontal pocket. BOP was measured at one site per tooth using papillary bleeding index by Mühlemann ^20^. The OHIS was calculated by adding the debris index and the calculus index at two sites per tooth ^21^.

Patient grouping was according to the classification of periodontal and peri-implantitis conditions ^22^. The severe periodontitis case category included stage 3 and stage 4 periodontitis cases with CAL ≥5 mm and PD ≥6 mm. The control group included gingivitis, stage 1 and stage 2 periodontitis cases of CAL <5 mm and PD ≤5 mm ^20, 23^.

The examiner’s reliability was verified with an interclass correlation coefficient (ICC) of 0.741 (95% CI 0.507–0.846) and 0.837 (95% CI 0.784–0.875), indicating good and excellent agreement for PD and CAL, respectively. The kappa value for PI and BOP ranged from 0.737 to 0.805, indicating substantial agreement.

Gingival crevicular fluid (GCF) was collected from four sites of the deepest periodontal pocket and randomly at one site of each quadrant in the healthy sulcus. Saliva and supragingival plaque were cleaned gently with sterile cotton pellets, separated with cotton rolls, and air-dried before sampling. A sterile paper point ISO 30 (Roeko, Germany) was inserted gently into the periodontal pocket until it reached the base of pocket depth with mild resistance and held for about 30 seconds. Bleeding sites were carefully avoided. The collected samples were pooled then put in a plastic tube containing 1 mL of sterile phosphate buffered saline. The vial was vortexed for about 1 min and then centrifuged for approximately 20 min at 3000 rpm. The supernatants were collected and samples stored at −20°C until further processing.

### DNA extraction and real-time PCR

After centrifugation, DNA was extracted from GCF using a commercial kit (QIAamp DNA Mini Kit, Qiagen, Germany) according to the instructions. NanoQuant Infinite ^®^ M200Pro was used to check for the presence and quantity of extracted DNA. The amount of EBV DNA in the samples was determined using the real-time PCR technique. The analyzed amount of EBV DNA in each sample was in a volume of 10 μL. The single-copy gene encoding the EBV nuclear antigen 1 (EBNA1) was the target sequence (EBNA1). A commercial kit (EBV/ISIN/100, GeneProof, Brno, CZ) was utilized using a real-time PCR LightCycler ^®^ 480 II (Roche Diagnostics GmbH, Mannheim, Germany). The kit has four standards containing an EBV calibrator from 10 ^1^ to 10 ^4^ copies of EBNA1 per μL. From linear regression to standard curves, quantification was conducted by extrapolation. The PCR conditions were set as directed by the manufacturer.

### Statistical analysis

The findings were analyzed using IBM SPSS v.23 (RRID:SCR_019096), for which an open-access alternative would be JASP ( https://jasp-stas.org; RRID:SCR_015823). The Chi-squared test was used to determine the association between EBV and periodontitis. A p-value of 0.05 or less was considered statistically significance.

## Results

In total, 118 subjects with mild-to-severe periodontitis, ranging from 18 to 66 years old, mean of 38.15 ± 14.4 years, were evaluated for periodontal status, including EBV DNA in the gingival crevicular fluid. Table 1 shows the demographic and clinical periodontal parameters.

**Table 1.** Demographic data and clinical parameters.

Demographic data	Case (n = 59)	Control (n = 59)	p-value
Age (years) ^a^, median (IQR)	49 (19)	24 (14)	0.000**
Male ^b^, n(%)	19 (27.5)	50 (72.5)	0.000**
Female, n(%)	40 (81.6)	9 (18.4)	
Non-smokers ^b^, n(%)	43 (58.1)	31 (41.9)	0.036*
Smokers, n(%)	16 (36.4)	28 (63.6)	
Clinical parameters			
PD (mm) ^a^, median (IQR)	1.87 (0.63)	1.44 (0.33)	0.000**
CAL (mm) ^a^, median (IQR)	2.60 (1.25)	1.48 (0.42)	0.000**
BOP, mean ± SD ^b^	1.47 ± 0.90	0.77 ± 0.62	0.000**
Plaque index ^a^, median (IQR)	1.24 (0.57)	0.88 (0.72)	0.001*
OHIS ^a^, median (IQR)	2.19 (1.25)	1.68 (1.28)	0.026*
Number of teeth ^a^, median (IQR)	24 (6)	27 (2)	0.000**

a Mann–Whitney test. b Continuity correction. c Independent t-test

EBV DNA was detected in 28.0% of all subjects, 37.3% of severe periodontitis cases, and 18.6% of mild periodontitis control cases. The highest EBV DNA level was 4.55 × 10 ^5^ copies/mL in severe periodontitis. No statistically significant difference in the detected EBV DNA was found between the case and control groups ( Table 2).

**Table 2.** Prevalence of EBV DNA in GCF samples.

Variable	Case (n = 59)	Control (n = 59)	p-value
EBV ^a^, n (%)	22 (66.7)	11 (33.3)	0.086

a Mann–Whitney test.

In Table 3 showed the association between clinical parameters of periodontitis and EBV DNA detection. It showed that the EBV DNA load was significantly associated with the plaque index and OHIS, but not with age, gender, and other clinical parameters of periodontitis. Although not statistically significant, patients with severe periodontitis had higher EBV DNA detection than those with mild periodontitis.

**Table 3.** Demographic data and clinical parameters in relation to detected EBV DNA.

Variable	Detected (n = 33)	Not detected (n = 85)	p-value
Age (years) ^a^, median (IQR)	35 (26)	36 (28)	0.990
Male ^a^, n (%)	24 (34.8)	45 (65.2)	0.080
Female ^a^, n (%)	9 (18.4)	40 (81.6)	
PD ^a^, median (IQR)	1.67 (0.71)	1.59 (0.53)	0.159
CAL ^a^, median (IQR)	2.03 (1.42)	1.86 (1.13)	0.078
BOP ^a^, median (IQR)	1.11 (1.01)	0.89 (1.07)	0.337
PI, mean ± SD ^b^	1.29 ± 0.44	1.04 ± 0.52	0.002*
OHIS ^a^, median (IQR)	2.39 (1.06)	1.70 (1.44)	0.001*
Number of teeth ^a^, median (IQR)	26 (6)	26 (5)	0.765

a Mann–Whitney test. b Independent t-test

## Discussion

Periodontal disease was initially assumed to be caused by bacteria but recent research have revealed that herpesviruses, particularly EBV, are also involved ^24, 25^. This hypothesis emerged because some periodontal diseases cannot be explained by bacterial activity alone ^4^. Our data do not support the idea that EBV plays a role in periodontitis, as some other research’ conclusions ^10, 12^. In cases of severe periodontitis, the prevalence of EBV detected at periodontal pockets was about 37.3% which was only slightly lower than in a previous study in India with 44.2% in aggressive periodontitis ^9^ lower than observed values for chronic periodontitis 72.5% in Iran ^6^; 74.49% in Indonesia ^26^; and 66–68% in Japan ^7, 8^.

Age, gender, and smoking status were associated significantly with periodontitis, so were the clinical parameters of periodontitis ( Table 1). The findings support a prior study that found smoking to be a risk factor for periodontitis in periodontal classification ^23^.

The detected EBV DNA was not significantly associated with periodontitis, age, gender, and clinical parameters (pocket depth, clinical attachment level, bleeding on probing) of periodontitis (p > 0.05). However, the increasing plaque index and OHIS were significantly associated with detected EBV DNA ( Table 3). This is in agreement with the findings of a previous study ^15^. Low rates of EBV detection could have various reasons including study methodology. In our study, the periodontitis group had a mean of clinical attachment level of 2.74 ± 0.92, while in other studies, a mean clinical attachment level of 6.7 ± 1.6 mm did not indicate an association between prevalence of EBV and periodontitis ^12^. Similarly, the probing depth was 1.9 ± 0.52 mm, well below the 5.8 ± 1.2 mm in the comparison study.

We analyzed the presence of EBV DNA by sterile paper points in 118 patients from gingival crevicular fluid (GCF) as done previously using paper strips ^15^. In other studies, EBV DNA has been detected from subgingival plaque with sterile paper point ^7, 8, 12, 26^ or curettage ^6, 10, 27^. GCF is only found in small amounts in healthy sulcus, but it increases 5.5-fold during inflammation, as demonstrated in experimental gingivitis ^28^. The resting void volume in GCF is highly dependent upon the pocket depth; as a pocket increases from 3 to 6 mm, the resting void volume increases by 50% ^29^. After 6 weeks posttreatment of phase-I periodontal therapy, the GCF level in periodontitis decreased as compared to baseline ^15^. Subgingival plaque was considered gold standard for periodontal EBV detection, that’s why GCF may be one reason for the poor detection rates. However, infrequently EBV DNA detection was also found in the subgingival plaque with curette technique in another study ^11^. In this study, we used GCF since it is non-invasive technique that can also detect the host immune response such as cytokines. A proposed mechanism for the EBV role suggests that periodontal pockets harbor EBV and contain excessive reactive oxygen species, which induce excessive development of receptor activator of nuclear factor κB ligand (RANKL) that activates osteoclasts ^30^.

The lowest positive amount of EBV DNA was 3.4 copies/mL. The range for mild periodontitis patients was up to 7.98 × 10 ^4^ copies/mL and for the severe periodontitis cases up to 4.55 × 10 ^5^ copies/mL. In a previous study in Japan on chronic periodontitis, the observed upper range of detected EBV DNA was about 10 ^3^–10 ^9^ copies/mL and an order of magnitude higher in deep pockets (PD ≥ 5 mm) than in shallower pockets (PD ≤3 mm) ^8^.

Another reason for the variability is the grouping of periodontitis that does not present a good contrast between cases and controls when healthy subjects are uncommon so that subjects with gingivitis and mild periodontitis provide the control group. As the recent classification of periodontitis does not differentiate between aggressive and chronic periodontitis, the severity of periodontitis is based on staging ^23^.

Furthermore, sensitive methods like nested PCR ^6, 7^ to identify EBV DNA have the risk of overestimating the result, while other methods like automated real-time PCR assays in previous studies found EBV DNA less frequently ^10– 12^, including in our investigation.

A previous study about EBV in another Indonesian population of Bandung, West Java, found all subjects seropositive for EBV with 75% detection rate in subgingival EBV DNA using the same technique of qRT-PCR ^26^. Some differences in environmental, ethnic, and immunogenetic factors may contribute but are not expected to be significant. Limitations of the study include the relatively small sample size so the study may have a limited power ^26^.

## Conclusions

Our findings showed no significant association between EBV and periodontitis, although in cases of severe periodontitis, EBV DNA was detected more frequently and at a higher maximum level than in cases of mild periodontitis. Gingival crevicular fluid is useful to check the EBV load using a real-time PCR technique.

## Data availability

### Underlying data

Harvard Dataverse: Underlying data for ‘Association of subgingival Epstein–Barr virus and periodontitis’. https://doi.org/10.7910/DVN/AR3Y00

This project contains the following underlying data: F1000_EBV_RawData_EIA_2021.csv

Data are available under the terms of the Creative Commons Zero “No rights reserved” data waiver (CC0 1.0 Public domain dedication).

## Consent

Written informed consent for publication of the patient details was obtained from the patients.
