# Supplementary material for: Association of subgingival Epstein–Barr virus and periodontitis
Source: F1000Res. 2021 Jun 28;10:414. Originally published 2021 May 24. [Version 2] doi: 10.12688/f1000research.52624.2 (PMC8278249; doi:10.12688/f1000research.52624.2)
Supplement: Peer review history

# Peer review: Reviewer response for version 2

**DOI:** 10.5256/f1000research.58041.r88310

**Published:** 2021-07-13

**Authors:** Muhammad Sohail Zafar

**Affiliations:** 1 Department of Restorative Dentistry, College of Dentistry, Taibah University, Medina, Saudi Arabia

Many thanks for the revision and responding to the comments.

Is the work clearly and accurately presented and does it cite the current literature?

Yes

If applicable, is the statistical analysis and its interpretation appropriate?

Yes

Are all the source data underlying the results available to ensure full reproducibility?

Yes

Is the study design appropriate and is the work technically sound?

Yes

Are the conclusions drawn adequately supported by the results?

Yes

Are sufficient details of methods and analysis provided to allow replication by others?

Yes

Reviewer Expertise:

Dentistry

I confirm that I have read this submission and believe that I have an appropriate level of expertise to confirm that it is of an acceptable scientific standard.

# Peer review: Reviewer response for version 2

**DOI:** 10.5256/f1000research.58041.r87685

**Published:** 2021-07-12

**Authors:** Shahida Mohd-Said

**Affiliations:** 1 Centre for Restorative Dentistry, Faculty of Dentistry, National University of Malaysia (UKM), Kuala Lumpur, Malaysia

A relevant and interesting study on the association of EBV and Periodontitis. Good and clear Introduction and justified reason for study.

 Well written and detailed Methods, with good number of patients and samples. Clearly explained Results; Distribution of microorganism is more suitable to be used than Prevalence, as the samples do not represent the bigger population. Good discussion with relevant literature to support.

 Nonetheless I think the implication of the study findings within the interested population would benefit the paper better, with emphasise on its impact to the current or future management of periodontitis and the wellness of the particular population in general. Sound conclusion.

Is the work clearly and accurately presented and does it cite the current literature?

Yes

If applicable, is the statistical analysis and its interpretation appropriate?

I cannot comment. A qualified statistician is required.

Are all the source data underlying the results available to ensure full reproducibility?

Partly

Is the study design appropriate and is the work technically sound?

Yes

Are the conclusions drawn adequately supported by the results?

Yes

Are sufficient details of methods and analysis provided to allow replication by others?

Yes

Reviewer Expertise:

Periodontology - Microbiology and Immunology Research, Natural Product Drug Discovery Research, Oral Health Service and Management Systems Research

I confirm that I have read this submission and believe that I have an appropriate level of expertise to confirm that it is of an acceptable scientific standard.

# Peer review: Reviewer response for version 1

**DOI:** 10.5256/f1000research.55925.r86020

**Published:** 2021-06-16

**Authors:** Muhammad Sohail Zafar

**Affiliations:** 1 Department of Restorative Dentistry, College of Dentistry, Taibah University, Medina, Saudi Arabia

I have reviewed the manuscript “Association of subgingival Epstein–Barr virus and periodontitis” submitted to F1000Research for publication. In this study, authors have investigated the potential relationship between Epstein–Barr virus and periodontitis. This is a well conducted study and the manuscript fits well within the scope of the journal; it needs some improvements; there are a few suggestions that authors may consider to improve it further:

 The use of English language is reasonable, however, there are a number of punctuation and grammatical errors; that should be corrected and rephrased using academic English for a better flow of text for reader.

 Authors should clarify that whether the STROBE checklist was used or not.

 Authors should mention more information regarding the non-respondents (i.e., how many individuals were willing to participant and how many were not?).

 Authors have used GCF in this study to measure EBV level. Is there any study that showed the use of saliva to measure the EBV?

 The introduction section is very brief and barely cover the rationale of the study and background of GCF in context. Authors should expand the introduction section a bit more adding GCF details. The following articles on GCF may be included (which I am a co-author):

- Human gingival crevicular fluids (GCF) proteomics: an overview." Dentistry Journal 5.1 (2017): 12 ^1^.
- Bacteriological Evaluation of Gingival Crevicular Fluid in Teeth Restored Using Fixed Dental Prostheses: An In Vivo Study." *International Journal of Molecular Sciences* 22.11 (2021): 5463 ^2^.

Please add; What was the main purpose of using GCF in this study?

Is the work clearly and accurately presented and does it cite the current literature?

Yes

If applicable, is the statistical analysis and its interpretation appropriate?

Yes

Are all the source data underlying the results available to ensure full reproducibility?

Yes

Is the study design appropriate and is the work technically sound?

Yes

Are the conclusions drawn adequately supported by the results?

Yes

Are sufficient details of methods and analysis provided to allow replication by others?

Yes

Reviewer Expertise:

Dentistry

I confirm that I have read this submission and believe that I have an appropriate level of expertise to confirm that it is of an acceptable scientific standard, however I have significant reservations, as outlined above.

## References

[1] : Human Gingival Crevicular Fluids (GCF) Proteomics: An Overview. *Dent J (Basel)* .2017;5(1) : 10.3390/dj5010012 10.3390/dj5010012 29563418PMC5806989

[2] : Bacteriological Evaluation of Gingival Crevicular Fluid in Teeth Restored Using Fixed Dental Prostheses: An In Vivo Study. *Int J Mol Sci* .2021;22(11) : 10.3390/ijms22115463 10.3390/ijms22115463 34067261PMC8196846

# Response

**Published:** 2021-06-22

**Authors:** Elza Ibrahim Auerkari

**Affiliations:** University of Indonesia, Indonesia

*"I have reviewed the manuscript “Association of subgingival Epstein–Barr virus and periodontitis” submitted to F1000Research for publication. In this study, authors have investigated the potential relationship between Epstein–Barr virus and periodontitis. This is a well conducted study and the manuscript fits well within the scope of the journal; it needs some improvements; there are a few suggestions that authors may consider to improve it further:*

*The use of English language is reasonable, however, there are a number of punctuation and grammatical errors; that should be corrected and rephrased using academic English for a better flow of text for reader."*

**Answer: We check again the punctuation and grammatical errors.**

*"Authors should clarify that whether the STROBE checklist was used or not."*

**Answer: STROBE checklist was not used in our study but our manuscript was checked against the STROBE checklist by the academic editor journal, and we completed the manuscript according to that checklist.**

*"Authors should mention more information regarding the non-respondents (i.e., how many individuals were willing to participant and how many were not?)."*

**Answer: We added in the non-respondents in the methods.**

*"Authors have used GCF in this study to measure EBV level. Is there any study that showed the use of saliva to measure the EBV?"*

**Answer: Yes, some study did measure EBV level in GCF or saliva or subgingival plaque. We added in the introduction.**

*"The introduction section is very brief and barely cover the rationale of the study and background of GCF in context. Authors should expand the introduction section a bit more adding GCF details. The following articles on GCF may be included (which I am a co-author):*

- *Human gingival crevicular fluids (GCF) proteomics: an overview." Dentistry Journal 5.1 (2017): 12 ^1^ .*
- *Bacteriological Evaluation of Gingival Crevicular Fluid in Teeth Restored Using Fixed Dental Prostheses: An In Vivo Study." International Journal of Molecular Sciences 22.11 (2021): 5463 ^2^ ."*

**Answer: Thank you for the suggestion, we added the references in the introduction section.**

*"Please add; What was the main purpose of using GCF in this study?"*

**Answer: We added in the purpose of using GCF in the discussion.**
